# Novel Filamin C Myofibrillar Myopathy Variants Cause Different Pathomechanisms and Alterations in Protein Quality Systems

**DOI:** 10.3390/cells12091321

**Published:** 2023-05-05

**Authors:** Dominik Sellung, Lorena Heil, Nassam Daya, Frank Jacobsen, Janine Mertens-Rill, Heidi Zhuge, Kristina Döring, Misagh Piran, Hendrik Milting, Andreas Unger, Wolfgang A. Linke, Rudi Kley, Corinna Preusse, Andreas Roos, Dieter O. Fürst, Peter F. M. van der Ven, Matthias Vorgerd

**Affiliations:** 1 Department of Neurology, Heimer Institute for Muscle Research, University Hospital Bergmannsheil, Ruhr-University Bochum, 44789 Bochum, Germany; dominik.sellung@bergmannsheil.de (D.S.); nassam.daya@rub.de (N.D.); frank.jacobsen@rub.de (F.J.); janine.mertens-rill@bergmannsheil.de (J.M.-R.); heidi.zhuge@rub.de (H.Z.); andreas.roos@uni-essen.de (A.R.); 2 Department of Molecular Cell Biology, Institute for Cell Biology, University of Bonn, 53121 Bonn, Germany; lheil@uni-bonn.de (L.H.); dfuerst@uni-bonn.de (D.O.F.); 3 Department of Human Genetics, Ruhr-University Bochum, 44801 Bochum, Germany; kristina.doering@rub.de; 4 Erich and Hanna Klessmann Institute, Heart and Diabetes Centre NRW, University Hospital of the Ruhr-University Bochum, 32545 Bad Oeynhausen, Germany; mpiran@hdz-nrw.de (M.P.); hmilting@hdz-nrw.de (H.M.); 5 Institute of Physiology II, University of Münster, 48149 Münster, Germany; aunger@uni-muenster.de (A.U.); wlinke@uni-muester.de (W.A.L.); 6 Department of Neurology and Clinical Neurophysiology, St. Marien-Hospital Borken, 46325 Borken, Germany; rudikley@t-online.de; 7 Department of Neuropathology, Charité—Universitätsmedizin Berlin, 10117 Berlin, Germany; corinna.preusse@charite.de

**Keywords:** myofibrillar myopathy, cardiomyopathy, filamin c, protein quality system, chaperone-assisted selected autophagy, protein folding

## Abstract

Myofibrillar myopathies (MFM) are a group of chronic muscle diseases pathophysiologically characterized by accumulation of protein aggregates and structural failure of muscle fibers. A subtype of MFM is caused by heterozygous mutations in the filamin C (*FLNC*) gene, exhibiting progressive muscle weakness, muscle structural alterations and intracellular protein accumulations. Here, we characterize in depth the pathogenicity of two novel truncating FLNc variants (p.Q1662X and p.Y2704X) and assess their distinct effect on FLNc stability and distribution as well as their impact on protein quality system (PQS) pathways. Both variants cause a slowly progressive myopathy with disease onset in adulthood, chronic myopathic alterations in muscle biopsy including the presence of intracellular protein aggregates. Our analyses revealed that p.Q1662X results in FLNc haploinsufficiency and p.Y2704X in a dominant-negative FLNc accumulation. Moreover, both protein-truncating variants cause different PQS alterations: p.Q1662X leads to an increase in expression of several genes involved in the ubiquitin-proteasome system (UPS) and the chaperone-assisted selective autophagy (CASA) system, whereas p.Y2704X results in increased abundance of proteins involved in UPS activation and autophagic buildup. We conclude that truncating *FLNC* variants might have different pathogenetic consequences and impair PQS function by diverse mechanisms and to varying extents. Further studies on a larger number of patients are necessary to confirm our observations.

## 1. Introduction

Myofibrillar myopathies (MFM) are severe, progressive hereditary neuromuscular disorders characterized by intramyoplasmic protein aggregations and focal dissolution of myofibrils [1,2,3]. Causative MFM variants have been identified in 10 genes: *DES* (desmin) [1], *CRYAB* (alpha-crystallin B chain) [2], *MYOT* (myotilin) [3], *LDB3* (LIM domain binding protein 3)/ZASP (Z-band alternatively spliced PDZ motif-containing protein) [4], *FLNC* (filamin-C) [5], *BAG3* (BAG family molecular chaperone regulator 3) [6], *FHL1* (four and a half LIM domains 1) [7], *TTN* (titin) [8], *DNAJB6* (DnaJ homolog subfamily B member 6) [9], and *HSPB8* (heat shock protein beta-8) [10]. These genes encode proteins associated with the Z-disc, a main structure serving vital roles in transmitting tension and contractile forces along the myofibrils and as a major signaling hub [11,12].

The FLNc-associated subtype of MFM, caused by heterozygous *FLNC* variants (MFM5; MIM# 609524), was discovered in 2005 [5], and subsequently an increasing number of families with MFM-filaminopathy were described [5,6,13,14,15]. The main symptoms are progressive skeletal muscle weakness, which usually occurs between the fourth and sixth decade of life, as well as respiratory insufficiency in the course of disease progression. MRI reveals a typical pattern of predominant lower limb muscle involvement helpful in differential diagnostics [16,17,18,19]. In addition, *FLNc* mutations may also lead to distal myopathy with histopathological features distinct from MFM [20,21,22,23]. More recently, an even larger number of *FLNC* mutations was shown to cause a range of isolated cardiomyopathies [13,24,25].

The *FLNC* gene maps to human chromosome 7q32.1 and encodes the predominant filamin isoform expressed in striated muscle cells. As with FLNa and FLNb, FLNc consists of an N-terminal actin-binding domain and 24 Ig-like domains that serve as versatile protein interaction interfaces. The carboxyterminal Ig-like domain 24 forms homodimers, enabling filamins to cross-link actin filaments [26,27], which is a crucial function of all filamins [18]. FLNc expression is required for sarcomere organization, maintenance, and physiological function [28]. It binds numerous Z-disc-associated proteins including myopodin/SYNPO2 [20], FATZ/calsarcin/myozenin [21,22,23], aciculin/PGM5 [29] and myotilin [30,31], while it interacts at the sarcolemma with components of the dystrophin-dystroglycan complex [32] and acts as scaffold for transmembrane receptors, as well as signaling and adapter proteins [33].

The cellular protein quality system (PQS) is essential for maintenance of skeletal muscle homeostasis and to maintain functional protein networks even under conditions that threaten protein integrity (e.g., under mechanical stress). PQS includes (macro)autophagy, the ubiquitin-proteasome system (UPS) and chaperone-assisted selected autophagy (CASA) and is thus regulated by a plethora of accessory proteins. Autophagy is mainly involved in the removal of unnecessary or dysfunctional components from the cell [34]. Protein degradation is mediated by the UPS or CASA. In both cases, protein clearance is usually initiated by the attachment of an ubiquitin-derived degradation signal to the target protein, involving the E1 ubiquitin-activating enzyme, one of a small number of E2 ubiquitin-conjugating enzymes, and a client-specific E3 ubiquitin-protein ligase (e.g., TRIM32). The target degradation signal is recognized either by ubiquitin-receptors in the proteasome or by autophagic ubiquitin adaptors. More specifically, actin-myosin contractions may cause force-induced unfolding of FLNc at the myofibrillar Z-disc. Mechanically unfolded and damaged FLNc is recognized by the BCL-2-associated athanogene 3 (BAG3) complex and directed to the CASA pathway for disposal [34].

Here, we characterize the clinical and histopathological phenotype of three patients with MFM-filaminopathy caused by two novel truncating *FLNC* variants. As disease control, a patient with the well-known pathogenic p.W2710X *FLNC* variant was included. Unexpectedly, our protein expression, transcriptomic and biophysical studies revealed distinct pathogenetic consequences and dysregulations in PQS.

## 2. Materials and Methods

### 2.1. Patients

Three German patients with MFM-filaminopathy from two families (I:1; II:1 and II:2) were included in this study. They underwent extensive neurologic examinations, and muscle MRI with a 1.5 Tesla MR unit (MAGNETOM Aera, Siemens, Munich, Germany) or a 3 Tesla MR unit (MAGNETOM Skyra, Siemens), respectively, following previously published standard protocols [16].

### 2.2. Methods

#### Standard Protocol Approvals, Registrations, and Patient Consents

Informed consent was obtained from all patients (approval of Ruhr-University Bochum ethics committee [#4368–121]).

### 2.3. FLNC Mutation Detection and Analysis of Mutant Allele Expression by RT-PCR

*FLNC* gene analysis by Sanger sequencing was performed according to an established protocol that avoids amplification of the *FLNC* pseudogene [35]. Variant detection was performed by aligning the derived sequence with the NCBI Reference Sequence NM_001458.4 using Gensearch (Phenosystems, Braine le Chateau, Belgium). In addition, for patient I:1, a whole-exome sequencing (WES) library was constructed using the Twist Comprehensive Exome/Twist Library Preparation Kit (V2) (Twist Bioscience) and sequenced as 2 × 150 nt paired end reads on a NextSeq1000 instrument (Illumina, San Diego, CA, USA). Sequence alignment, variant calling, and annotation were performed using the Varvis bioinformatics pipeline v1.20 (Limbus Medical Technologies GmbH, Rostock, Germany). Detected variants were evaluated based on the ACMG classification system [36] and the phenotype of the patient.

For analysis of the expression level of both *FLNC* alleles in our patients at the RNA level, total RNA was isolated from muscle biopsies using the RNeasy fibrous tissue mini kit according to the instructions of the manufacturer (Qiagen, Hilden, Germany). cDNA was prepared using random nonamers and the FIREScript RT cDNA Synthesis Kit (Solis Biodyne, Tartu, Estonia). cDNA was amplified by PCR using oligonucleotides TTCGTGGGCCAGAAGAACT and TGTAGCCACCAGAGAGACCA (p.Y2704X) or TCCGCATCCATGCTCTGCCCACTG and CTTGGCATCCACCGTGATCAGCGTCTCC (p.Q1662X) using FIREPol Master Mix Ready to Load (Solis Biodyne). The latter primer contains a single mismatch introducing a HpyF10VI restriction site in the wildtype but not the mutant amplicon. To discern between both variants, the resulting amplicons were purified and digested with the restriction enzyme BseGI (that digests the normal variant but not the p.Y2704X mutant) or HpyF10VI (that digests the normal variant but not the p.Q1662X mutant). Fragments were analyzed by agarose gel electrophoresis. Gels were photographed using a GelDoc XR Imaging system (Bio-Rad, Feldkirchen, Germany), and bands were quantified using densitometry (QuantityOne software, Bio-Rad).

### 2.4. Histochemistry and Immunohistochemistry

Skeletal muscle biopsies from patients I:1 (from soleus muscle) and II:2 (from vastus lateralis) were used for evaluating histopathological changes, immunolocalization and electron microscopy studies, using established procedures [16,37].

Double immunofluorescence staining was performed on 6 µm frozen serial sections of muscle biopsies with the primary and secondary antibodies listed in Supplemental Table S1. 

### 2.5. Cloning of the Truncated p.Y2704X FLNc Construct, and Expression and Purification of Recombinant Proteins

A FLNc d23–24 construct containing the p.Y2704X variant was obtained by PCR using primers tttacgcgtGGGGAGCAGAGCCAGGCTGGGGACCCAG (forward) and tttgtcgacGTCCCCTTTCTCCTTGACAGT (reverse) and the wild-type variant as a template. The amplicon was digested with MluI and SalI, and cloned into the prokaryotic expression vector pET23-T7 [38] to enable expression of a recombinant protein carrying an amino-terminal T7-immunotag and a carboxyterminal His_6_-tag. The integrity of the construct was verified by sequencing (LGC Genomics, Berlin, Germany). The construct was transformed to *E. coli* BL21(DE3)CodonPlus cells (Stratagene, La Jolla, CA, USA). Protein expression and purification of His6-tagged proteins were performed as described [26,39].

### 2.6. Cross-Linking of FLNc Polypeptides

The dimerization property of mutant p.Y2704X FLNc was examined by chemical cross-linking experiments, using previously established protocols [26,39]. In brief, purified recombinant His6- and EEF-tagged wild-type and His_6_- and T7-tagged p.Y2704X FLNc fragment (Ig-like domains 23–24) were incubated in the absence or presence of the cross-linker ethylene glycol bis(succinimidyl succinate) (EGS). Formation of cross-linked dimers and aggregates, that are not separated by boiling in SDS-sample buffer, was analyzed by SDS-PAGE and Western blotting using specific antibodies against the respective immunotags of normal and mutant FLNc. Blots were photographed using a ChemiDoc MP Imaging System (Bio-Rad).

### 2.7. Proteolytic Susceptibility Studies

Proteolytic susceptibility was investigated using thermolysin (Sigma, St. Louis, MO, USA) essentially as described [39]. To 140 μL recombinant protein (10 μM in 50 mM NaH_2_PO_4_, 300 mM NaCl, 250 mM imidazole, pH 8.0), 7 μL thermolysin (0.1 mg/mL) was added and the mixture was incubated at 37 °C. At different incubation intervals, the reaction was stopped by adding 0.2 vol. 5× SDS sample buffer. After heating at 95 °C for 5 min, 10 μL of each sample was analyzed by SDS-PAGE using 14% polyacrylamide gels. Gels were photographed using a ChemiDoc MP Imaging System (Bio-Rad).

### 2.8. Transcript Studies on cDNA of Patient Skeletal Muscle Biopsies

Total RNA was extracted from skeletal muscle biopsies using a Trizol-chlorofom method, followed by cDNA transcription using the High-Capacity cDNA Archive Kit (Applied Biosystems, Foster City, CA, USA). For qPCR reactions, 10 ng of cDNA were used. Subsequent analysis was performed on the QuantStudio 6 Flex System (Applied Biosystems, Foster City, CA, USA) with the following running conditions: 95 °C 20 s, 95 °C 1 s, 60 °C 20 s, 45 cycles (values above 40 cycles were defined as “not expressed”). All targeted transcripts were run as triplicates. For each of these runs, the reference gene *PGK1* was included as internal control to normalize the relative expression of the targeted transcripts. The qPCR assay identification numbers, TaqMan^®^ Gene Exp Assay from Life Technologies/ThermoFisher are as follows: BAG3 HS00188713_m1, BECN1 Hs00186838_m1, CALR Hs00376764_m1, HSPA5 (Grp78)/BiP Hs00607129_gH, HSPA8 Hs03044880_gH, LAMP1 Hs00931461_m1, LC3/MAP1 Hs01076567_g1, LMP7 (PSMB8) Hs00544760_g1, PSME1 Hs00389210_g1, PSME2 Hs01923165_u1, PGK1 Hs99999906_m1, SQSTM/p62 Hs01061917_g1. Gene expression is visualized as fold change (2^−ΔΔCT^) to non-disease controls (NDC).

### 2.9. Western Blotting of Control and Patient Samples

To analyze the expression levels of FLNc and markers of the PQS machinery (SQSTM1/p62, TRIM32 and BAG3), 20 cryosections (10 µm thickness) prepared from human muscle biopsies (n = 2 normal controls, n = 2 patients with sporadic inclusion body myositis (sIBM included as further disease control based on the well-known activation of the PQS), p.Q1662X, p.Y2704X, p.W2710X) were lysed in 100 mM Tris-buffer (pH 8.6) containing 2 M thiourea, 7 M urea, 5 mM EDTA, 1 mM dithiothreitol and 5µL/mL protease-inhibitor-mixture (oComplete, Merck, Darmstadt, Germany) for 15 min at RT. Samples were sonicated (1 min, 1 W, 20% amplitude) to enhance lysis. 2× Lämmli-buffer (4% *w*/*v* SDS, 20% *v*/*v* glycerol, 10% *v*/*v* beta-mercaptoethanol, 0.004% *w*/*v* bromophenol blue and 125 mM Tris/HCl pH 6.8) was added and 10 µL of each sample was loaded together with a molecular weight marker on a 4–20% polyacrylamide gel (4–20% Mini-Protean TGX precast, Biorad, Feldkirchen, Germany) and run at 180–200 V using a Biorad Tetracell-System. Proteins were transferred (Trans-Blot^®^ SD Semi-Dry Transfer Cell, Bio-Rad, CA, USA) (transfer buffer: 25 nM Tris, 190 mM glycin and 20% *v*/*v* methanol) to an activated PVDF-membrane for 50 min at 12 V. Membranes were blocked in PBS-T containing 5% *w*/*v* BSA at 4 °C over night. Primary and secondary antibodies were diluted in PBS-T containing 5% *w*/*v* BSA and incubated for at least 1 h at room temperature. In between and afterwards, blots were washed three times in PBS-T. Subsequently, ECL-solution was pipetted on the membrane and the corresponding light emission signals were detected by a CCD-Camera system (Azure c600, Azure Biosystems, Dublin, CA, USA). Densitometry was performed using AzureSpot Pro (Azure Biosystems, Dublin, CA, USA). Specific signals were normalized to the total amount of detectable proteins determined by a corresponding Coomassie-stained polyacrylamide gel.

For Western blot analyses using different FLNc antibodies, protein extracts prepared from approximately 25 mg of a skeletal muscle biopsy as described [40], were separated on a polyacrylamide gel and transferred onto nitrocellulose membranes using a Mighty Small transfer tank (Hoefer, Holliston, MA, USA). Membranes were incubated with primary antibodies and horseradish peroxidase (HRP)-conjugated secondary antibodies (Jackson ImmunoResearch). Signals were detected using SuperSignal West Pico PLUS Chemiluminescent Substrate and documented using a ChemiDoc MP Imaging System (Bio-Rad). Densitometric analysis was performed using Image Lab version 6.1.0 software (Bio-Rad). Signals were normalized to the expression level of α-tubulin.

## 3. Results

### 3.1. Identification of Two Novel FLNC Variants

**Figure 1:**
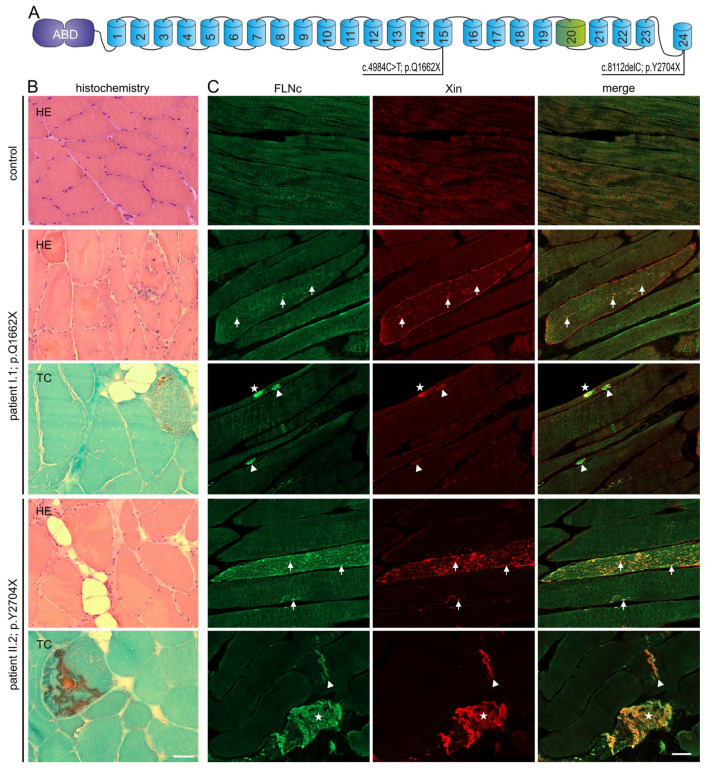
Genetic and histochemical findings and immunofluorescence studies. (**A**) Schematic presentation of the FLNc protein with the aminoterminal actin-binding domain (ABD, purple) and 24 Ig-like domains (blue). Domain 20 (green) is colored differentially because it is extended and contains an 82 amino acid long insertion. The locations of the two novel mutations are shown. (**B**,**C**) Analysis of muscle biopsy samples from control, patients I:1 (p.Q1662X) and II:2 (p.Y2704X). (**B**) Cryosections stained with H&E and trichrome (TC) showed fiber diameter variability, fiber splitting, fatty replacement, endomysial fibrosis and increase in central nuclei. These findings were more pronounced in II:2 (p.Y2704X). Scale bars: 50 µm. (**C**) Immunolocalization of FLNc and muscle damage marker Xin in longitudinal cryosections of control and patient muscle. FLNc and Xin co-localize in protein aggregations (asterisk), macrolesions (arrowheads) and microlesions (arrows) in patients I:1 and II:2, but not in control muscle fibers. Scale bars: 50 µm.

We describe three affected patients with MFM-filaminopathy from two unrelated families. Sanger sequencing of the FLNC gene of index patient I:1 revealed a novel variant (c.4984C > T) that results in a premature stop codon at amino acid position 1662 (p.Q1662X) which is located in Ig-like domain 15 (Figure 1A). Whole-exome sequencing confirmed this heterozygous mutation and did not reveal any other known or potential pathogenic variant. In the other index patient (II:2) and her affected mother (II.1), a heterozygous deletion mutation (c.8112delC) was found that leads to a premature truncation of the protein (p.Y2704X) in domain 24 (Figure 1A). Similar to the first described pathogenic FLNc variant (p.W2710X) [5], the p.Y2704X mutation results in a truncation of the carboxyterminal dimer-forming FLNc Ig-like domain 24, in this case of 22 amino acids.

### 3.2. Clinical Features

Disease onset in the male p.Q1662X patient (I:1) was at the age of 57 years. Initial symptoms were distal lower leg muscle weakness and atrophy. At 60 years of age clinical examination showed slight distal leg muscle weakness of 4/5 MHC grade and atrophy of the calf muscles. He moreover presented a coronary artery disease with a previously diagnosed myocardial infarction at 60 years of age, and a reduced, 65% forced vital capacity (FVC), indicating a moderate respiratory insufficiency. In the female index patient of the second *FLNC* family (p.Y2704X, patient II:2), the onset was at the age of 53 years. Here, the most prominent clinical symptom was proximal lower leg muscle weakness without sensory symptoms. Clinical examination revealed weakness of proximal upper and lower limb muscles of MHC grade 4/5. Her mother (II:1) reported initial weakness of lower limb and axial muscles and a slowly progressive disease course with additional upper limb weakness. Examination at the age of 77 years showed pronounced weakness in her upper and lower limb muscles of grade 3/5 and she used a wheelchair. Creatine kinase values in patient I:1 were 221 U/L (normal < 172 U/L), in patient II:1 325 U/L and in patient II:2 501 U/L (normal < 142 U/L).

### 3.3. Histopathological and Ultrastructural Studies

Analysis of a muscle biopsy obtained from patient I:1 (p.Q1662X) revealed myopathic features including with fiber size variability of type 1 and type 2 fibers, numerous split muscle fibers, central nuclei in 30% of the fibers, focal increased fibrous and fatty connective tissue, and a single ragged red fiber in trichrome (TC) staining (Figure 1B). Patient II:2 (p.Y2704X) also presented with myopathological features including with fiber size variation, fiber splitting, centrally located nuclei in 30%, and small rimmed vacuoles in some muscle fibers. TC showed muscle fibers with a combination of vacuolar changes and blue-colored amorphous deposits (Figure 1B). Immunolocalization studies revealed a few FLNc positive aggregations within abnormal muscle fibers in patient I:1, and numerous intracellular aggregations in patient II:2 (Figure 1C). To discern between aggregates and lesions, we also performed immunolocalization studies using antibodies against FLNc and the muscle damage marker Xin on longitudinal sections. This approach revealed a few FLNc and Xin positive micro- and macrolesions, and small aggregates within abnormal muscle fibers in patient I:1, and numerous lesions and large aggregates in patient II:2 (Figure 1C).

**Figure 2:**
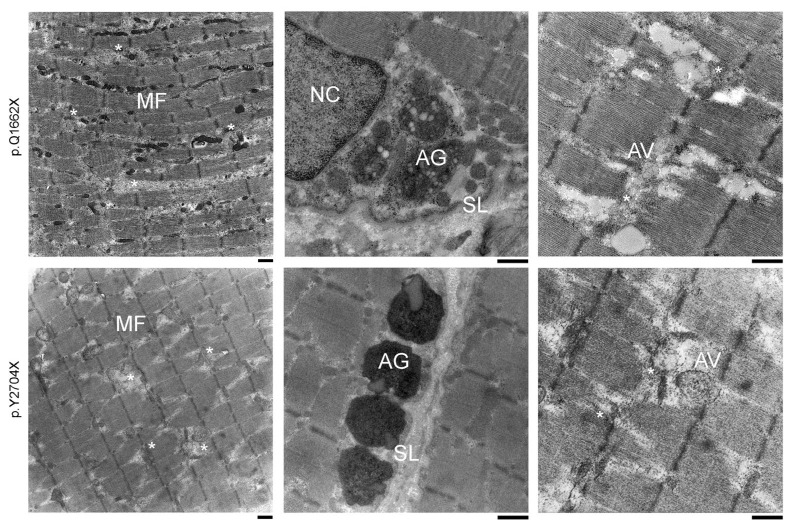
Electron microscopy showing ultrastructural overview of pathologic hallmarks. (**left panels**) Ultrastructural analysis of muscle biopsy samples from patients I:1 (p.Q1662X) and II:2 (p.Y2704X) showing both typical signs of MFM pathology. Extended areas of myofibrillar disorganization with sarcomeric lesions and (**central panels**) electron-dense material connecting adjacent Z-discs surrounded by many autophagic vesicles. (**right panels**) Frequent observation of subsarcolemmal depositions including heterogeneous, granulofilamentous protein aggregates (*) mainly localized at the subsarcolemmal level and in the perinuclear environment. MF: myofibril, AG: aggregates, NC: nucleus, SL: sarcolemma, AV: autophagic compartment, asterisks: degradation area, Scale bar: 1 μm.

Electron microscopical analysis of patient I:1 (p.Q1662X) and patient II:2 (p.Y2704X) showed clear pathological alterations in the ultrastructure of the contractile apparatus (Figure 2). Both phenotypes revealed large-scale degradation of myofibrils, intersarcomeric lesions and fracture lines with a massive presence of autophagic vesicles. In the p.Q1662X patient biopsy, myocytes showed many dissociated sarcomeres, some myocytes were completely degraded. The p.Y2704X biopsy revealed local degenerations of the sarcomeres. In both phenotypes, the disorder seemed to originate from or nearby the Z-disks. Both biopsies revealed MFM-typical aggregates. These polymorphic, granulovesicular protein conglomerates are mainly located subsarcolemmal or in perinuclear areas of the altered myocytes.

### 3.4. Magnetic Resonance Imaging (MRI) Studies

**Figure 3:**
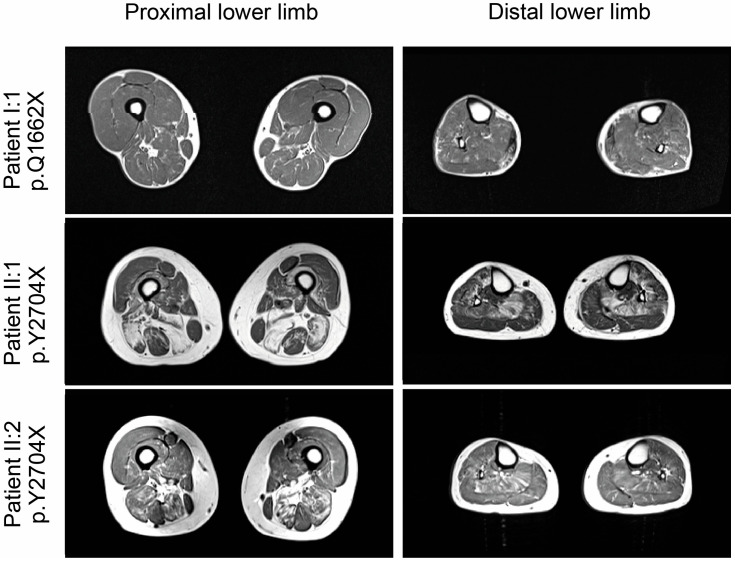
Muscle imaging findings in lower extremities.T1-weighted muscle MRI revealed no major alterations in patient I:1 (p.Q1662X), and moderate fatty degenerative changes in posterior thigh muscles (especially semimembranosus and biceps femoris), soleus and anterior tibial muscles in patients II.2 and II:1 (both p.Y2704), which were pronounced in the older patient (II:1).

MRI in patient I:1 showed normal thigh and calf muscles on T1-weighted images, but edematous alterations of the soleus muscles on STIR-images. (Figure 3) Patient II:1 displayed symmetrical fatty degenerative changes in posterior thigh muscles (especially semimembranosus and biceps femoris), soleus and anterior tibial muscles. A reticular pattern of hyperintensity on T1-weighted images was observed in the semitendinosus and in the medial head of the gastrocnemius, the extensor hallucis longus and extensor digitorum longus muscles (Figure 3). Patient II:2 showed a similar pattern of alterations in the posterior thigh muscles, but to a lesser degree. In the lower legs, reticular fatty degeneration was observed in the soleus muscles (Figure 3).

**Figure 4:**
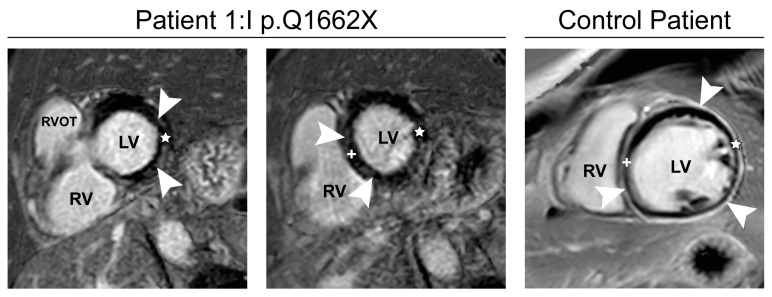
Cardiac MRI. Phase-sensitive inversion-recovery (PSIR) of patient I:1, Basal (**left**) and mid-short-axis (**middle**) images show subepicardial to mid-wall late enhancement (arrowheads) in the lateral wall (star) and interventricular septum (+). Typical example late gadolinium enhancement image with diffuse late enhancement in a 23-year-old control patient with FLNC-cardiomyopathy (p.E2009GfsX29; (**right**), arrowheads). RVOT: Right ventricular outflow tract, LV: Left ventricle, RV: Right ventricle.

Cardiac MRI in patient I:1 with MFM-filaminopathy showed subepicardial to mid-wall late enhancement in the lateral wall and interventricular septum (Figure 4). This pattern is clearly different from ischemic cardiomyopathies and typically seen in *FLNC*-related cardiomyopathies (FLNc control patient in Figure 4). Therefore, we suggest that FLNc patient I:1 presents with an overlap of early skeletal muscle and cardiac involvement.

### 3.5. Expression Analysis of Mutant and Normal FLNC RNA and FLNc Protein

**Figure 5:**
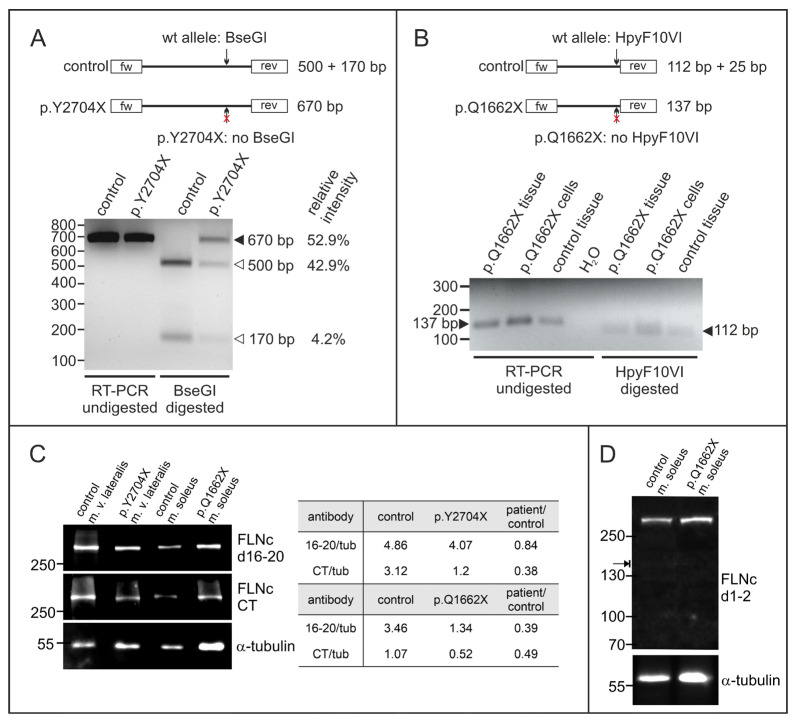
Analysis of the expression of the mutant alleles at protein and mRNA level. (**A**) RT-PCR analysis of the p.Y2704X patient. Specific primers were used to amplify a 670 bp long fragment of FLNc cDNA containing the mutation. Digestion with restriction enzyme BseGI cuts the normal (arrow) but not the mutant variant (arrow with red ×). In control cDNA, only 500 bp and 170 bp fragments, but no non-digested fragment was detected (670 bp), while the relative intensity measured by densitometry indicated that ~53% of the p.Y2704X cDNA is not digested, and thus mutant. (**B**) RT-PCR analysis of p.Q1662X patient tissue and cultured myocytes. Specific primers were used to amplify a 137 bp long fragment of FLNc cDNA containing the mutation (arrow on the left). Digestion with restriction enzyme HpyF10VI cuts the normal (arrow) but not the mutant variant (arrow with red×). In control as well as patient cDNA, only digested 112 bp fragments were found (arrow on the right), while no non-digested fragment was detected at 137 bp, indicating that no mutant mRNA is expressed in the patient. The 25 bp fragment is too small to be visible on the gel. (**C**) Analysis of FLNc protein expression in patient and control skeletal muscle tissue using an antibody specific for a central region of FLNc (d16–20) and its extreme C-terminus (CT). The relative expression level of FLNc when compared to the α-tubulin loading control indicates that in our p.Y2704X patient 84% of the normal FLNc level is expressed. The anti-CT antibody that does not recognize the p.Y2704X variant, detects only 38% of the level in normal vastus lateralis muscle. In p.Q1662X patient tissue, the level of FLNc recognized by both antibodies is decreased to 40–50% of the level in control tissue. (**D**) Total protein extracts from skeletal muscle biopsies from our p.Q1662X patient and control tissue were analyzed for the expression of FLNc derived from the wildtype allele (~291 kDa) and the mutant allele (~171 kDa, arrow) using an FLNc antibody recognizing the N-terminus (d1–2) of FLNc. Note that no extra band was observed in the extract from patient tissue, indicating no detectable levels of truncated protein expressed from the mutant allele.

For variants causing the occurrence of a premature stop codon it is crucial to investigate potential degradation of mutant mRNA via nonsense mediated decay (NMD). We therefore performed RT-PCR with mRNA purified from control and patient skeletal muscle specimens. At the cDNA level, the mutation leads to the deletion of a BseGI restriction site in the p.Y2704X variant, and a deletion of a HpyF10VI site in the p.Q1662X variant, enabling us to differentiate between the amplicons derived from the wild-type and mutant alleles. p.Y2704X patient cDNA exhibited both, non-digested mutant (670 bp) and digested wild-type (170 and 500 bp) cDNAs at a ratio of approximately 1:1 (~53% vs. ~47%) (Figure 5A), indicating that mutant mRNA is not degraded via NMD and that both wild-type and mutant RNAs are expressed, resulting in the expression of p.Y2704X-mutant FLNc protein in addition to wild-type FLNc. In contrast, in the p.Q1662X patient, the restriction enzyme digest using HpyF10VI resulted in complete digestion, indicating that mRNA encoding the p.Q1662X mutant is not expressed at a detectable level but instead is degraded by NMD (Figure 5B).

We analyzed the amount of FLNc protein in skeletal muscle biopsies of both patients compared to the amount of α-tubulin. Since FLNc levels vary significantly between different muscles, we used biopsy material from the same muscle from individuals without a muscle pathology as control. In the patient carrying the p.Y2704X mutation, staining with our FLNc antiserum raised against domains 16–20 indicated similar FLNc levels (84% of control), whereas the antiserum against the carboxy-terminus (CT) of FLNc, that is missing in the p.Y2704X mutant, revealed highly reduced levels (38% of control). By contrast, in the p.Q1662X patient, both antibodies showed a reduced expression of filamin C (39% and 49% of control for anti-filamin C d16–20 and filamin C CT, respectively) (Figure 5C). To investigate whether in the p.Q1662X patient a FLNc variant truncated at amino acid 1662 is expressed (predicted molecular mass 171 kDa), immunoblot using an antibody raised against FLNc domains 1–2 that should recognize this variant were performed. Clearly, no truncated FLNc was detected (Figure 5D). These results indicate a highly reduced level of normal FLNc in the muscle fibers of our p.Q1662X patient, whereas the total FLNc amount in the p.Y2704X patient is only slightly reduced compared to the normal level.

### 3.6. Analysis of Stability and Dimerization Capability of the p.Y2704X Variant Protein

**Figure 6:**
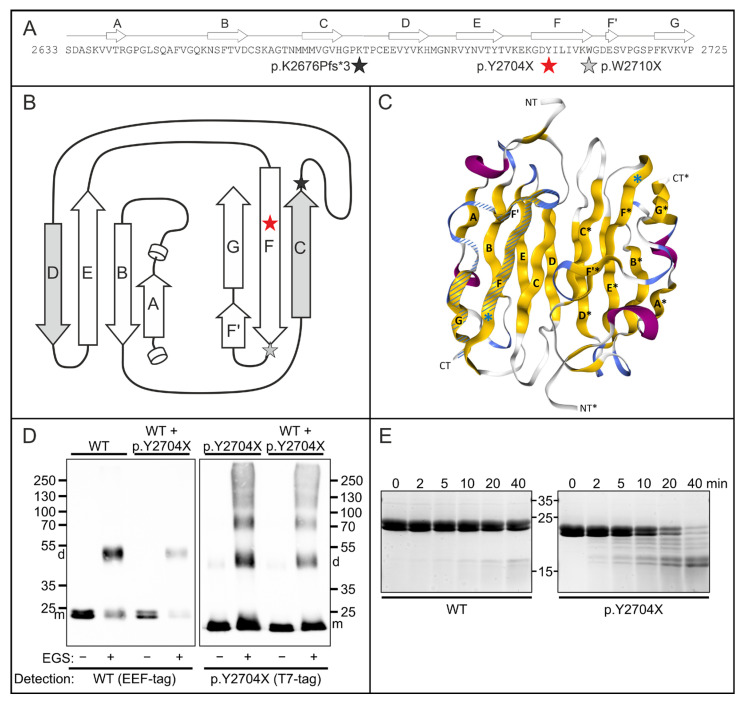
Functional characterization of the p.Y2704X mutation. (**A**) Schematic illustrations of the effect of the p.Y2704X mutation. Topology of filamin C Ig-like domain 24 aligned to the amino acid sequence. The position of the mutation is marked with a red asterisk. Grey and black asterisks in panels A and B mark previously found mutations in this domain. (**B**) Two-dimensional view of domain 24. The interface between dimerized domains is formed by β-strands C and D form. In p.Y2704X, FLNc strand G is lacking together with strand F’ and a part of strand F. Strand G normally interacts with strand A, which is important for the coherence of the β-sheets comprising an Ig-like domain. (**C**) Three-dimensional view of an FLNc domain 24-dimer (PDB code: 1V05). β-strands (yellow) are numbered as in panels A and B (A–G for one monomer, and A’–G’ for the second monomer). The premature stop codon in p.Y2704X is shown by a blue asterisk. The part of the domain that is deleted is shown hatched in one of the monomers. (**D**) Chemical cross-linking experiments using EEF-tagged wild-type (WT) and T7-tagged p.Y2704Xfilamin fragments (domains 23 + 24), or a mixture of both polypeptides (WT + p.2704X as indicated above the panels. Assays were performed in the absence (−) or presence (+) of the cross-linker EGS. Blots were either stained with antibodies detecting the EEF-tag of WT FLNc d23–24 monomers (m) and dimers (d), or the T7-tag of the p.2704 variant as indicated. Note normal dimerization of the wild-type protein, also in the presence of the mutant variant, whereas p.Y2704X FLNc also forms large, aggregated oligomers. (**E**) Protein stability analysis. Wild-type and p.Y2704X filamin C fragments (d23–24) were treated with the protease thermolysin for 0 to 40 min, as indicated. Samples were analyzed by polyacrylamide gel electrophoresis. The mutant protein was already partially digested after 2 min, and the largest part of the protein after 40 min, while the wild-type variant was still almost completely intact after 40 min. This indicates less stable folding of the mutant protein.

Since other pathogenic variants affecting the dimerization domain of FLNc (Figure 6A,B) had a marked effect on the ability of the protein to dimerize, we analyzed the capability of a carboxy-terminal fragment of mutant p.Y2704X FLNc to dimerize by chemical cross-linking experiments. While the wild-type protein clearly forms dimers, the corresponding truncated mutant protein (Figure 6C) exhibits rather inefficient dimer formation. Instead, a major portion of the cross-linked polypeptides is found in higher molecular mass oligomers and aggregates (Figure 6D). Mixtures of wild-type and mutant proteins did not change their respective properties, i.e., the wild-type protein still formed dimers in the presence of mutant protein, whereas the mutant protein continued to aggregate (Figure 6D). These experiments indicate that wild-type FLNc cannot restore the severely limited dimerization capability of the mutant protein.

We moreover examined effects of the p.Y2704X mutation on FLNc folding and stability using thermolysin digestion of d23–24 constructs. This assay is configurated in such a way that the protease should have little effect on a stably folded protein, whereas digestion of the protein would indicate altered or lacking stable folding. Accordingly, the analysis of the thermolysin-digested wild-type protein by polyacrylamide gel electrophoresis demonstrated that the protease has very little effect for as long as 40 min. In contrast, the mutant p.Y2704X protein began to show partial digestion already after 2 min, and most of the protein was digested after 40 min (Figure 6E). These results indicate a notably less stable fold of the mutant protein with many exposed and unfolded regions which are highly susceptible to proteolysis.

### 3.7. Transcript Studies on RNA Extracted from Human Muscle Biopsies

**Figure 7:**
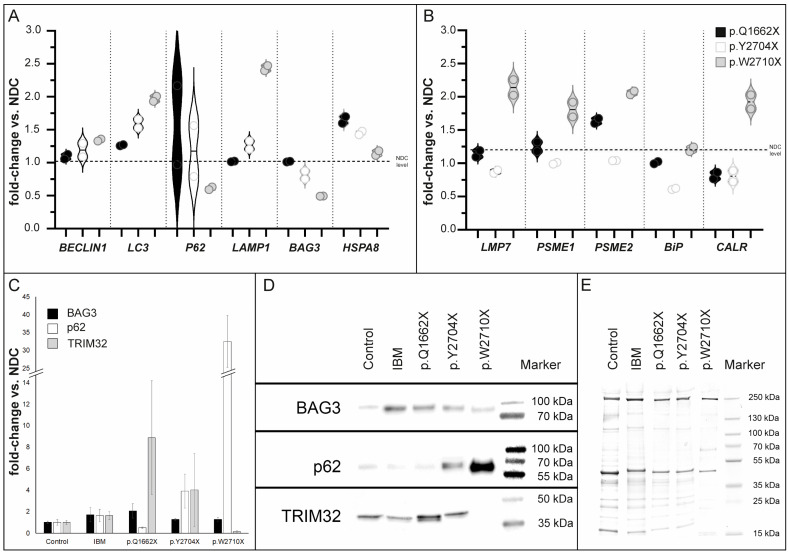
Transcript studies and immunoblot analysis of PQS markers in skeletal muscle tissue. (**A**) We have analyzed multiple genes involved in autophagy-, proteasome- and unfolded protein response (UPR) -pathways. The mutations differentially affect gene expressions, with upregulation of, e.g., LC3 in the FLNc variant p.W2710X. However, both new mutations do not show strong dysregulation of autophagy markers on the RNA level. In contrast, the patient with the p.Q1662X mutation showed increased HSPA8 expression. (**B**) Furthermore, upregulation of UPR markers and proteasome-associated genes, compared with non-disease controls (NDC), which was observed in the p.W2710X mutant, was not detected in patients with p.Q1662X or p.Y2704X mutation. (**C**) Densitometric data of the western blot signals were normalized to the corresponding total protein amount determined from coomassie based total protein stain. Values are shown in relation of the signals of the patients (IBM n = 2, p.Q1662X, p.Y2704X and p.W2710X) and the values of the non-disease controls (n = 2), respectively. Analysis was performed in technical triplicates. (**D**) Representative protein of interest bands together with the corresponding marker bands are shown. (**E**) A representative Coomassie-stained SDS-polyacrylamide is displayed to verify the total protein amount for each protein sample. Error bars indicate the deviation of the arithmetic mean of independent experiments.

The increase in FLNc-immunoreactive aggregates in both novel *FLNC* variants suggested that PQS pathways could be activated in these mutants. To study proteolysis, in addition to chaperones (BAG3, BiP, CALR, HSPA8) and ubiquitin along with ubiquitination related proteins (CHIP/STUB1 and TRIM32), well-known marker of autophagosome maturation (BECN1, FYCO1, LAMP1, LC3 and SQSTM1/p62) and proteasomal function (LMP7, PSME1 and PSME2) were analyzed. This also included the study of gene expression of multiple genes involved in autophagy, unfolded protein response (UPR) and proteasome pathways. The individual mutations lead to different variations in gene expression, with upregulation of *LC3* in the FLNc variant p.W2710X (Figure 7A). However, both new mutations do not show strong dysregulation of autophagy markers on the RNA level (Figure 7A). Interestingly, the clear upregulation of UPR markers *CALR* and *LAMP1* observed in the p.W2710X mutant, was not seen in patients I:1 (p.Q1662X) and II:2 (p.Y2704X) (Figure 7B). Furthermore, both new mutations do not cause an upregulation of the expression of proteasome-associated genes (Figure 7B), which was clearly seen in the p.W2710X patient. In contrast, patient I:1 showed increased *HSPA8* expression. Statistical analyses were not possible due to the small sample size and associated lack of repetition.

### 3.8. Analysis of Protein Expression of PQS Markers

Interestingly, semi-quantitative Western blot analysis of p.Q1662X muscle tissue lysates showed that the level of BAG3 and TRIM32 were increased, whereas a lower abundance of p62 expression was found. Analysis of the p.Y2704X muscle lysate, however, revealed increased levels of p62 and TRIM32 and a minor increase in BAG3. We infer that both novel *FLNC* variants might be associated with an upregulation of the UPS pathway, indicated by these involved proteins, whereas the autophagic system might be differently regulated: increased in p.Y2704X but downregulated in p.Q1662X. Interestingly, in the lysate of a biopsy specimen of the FLNc disease control p.W2710X, which contained a higher number of intracellular protein aggregates, p62 expression level was drastically increased but TRIM32 was less abundant (Figure 7B). In summary, these findings hint at dysregulation of the autophagy/UPS machineries.

### 3.9. Intracellular Distribution of PQS Markers

**Figure 8:**
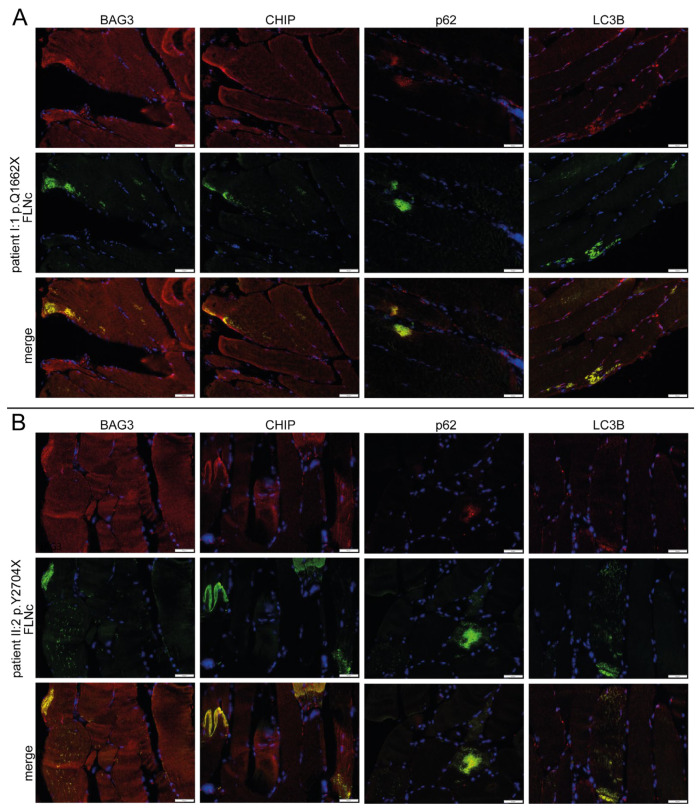
Immunolocalization studies of UPS markers. (**A**) Analysis of the distribution of CASA markers BAG3 CHIP, p62 and LC3B in double stained cryosections of muscle biopsies from patient I:1 (p.Q1662X) and (**B**) II:2 (p.Y2704X) using antibodies against FLNc (green) to localize aggregates and microlesions together with the aforementioned UPS-marker proteins (red). Scale bars: 50 µm.

As in our previous proteomic studies, immunolocalization studies revealed colocalization and accumulation of the chaperone-assisted selective autophagy (CASA) complex proteins BAG3, ubiquitin and CHIP in FLNc-immunoreactive protein aggregates as well as in microlesions in muscle biopsies from patients I.1 and II.2 (Figure 8) [37]. The macroautophagy- or aggrephagy-associated proteins LC3B and p62 showed the same localization (Figure 8), whereas FYCO1 as a positive regulator of autophagosome maturation [41] did not co-localize in these aggregates.

## 4. Discussion

**Figure 9:**
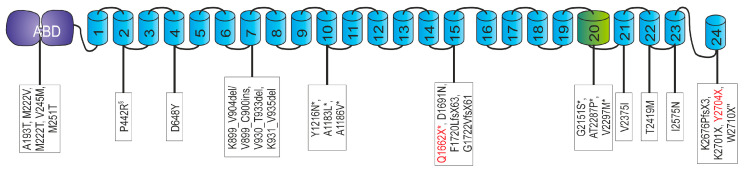
Overview of *FLNC* mutations causing isolated myopathy or combined myopathy and cardiomyopathy. Disease-associated variants in *FLNC* were found throughout the whole protein. In the actin-binding domain (ABD) [43,44], and also in domain 2 [42], domain 4 [45], domain 7 [46,47,48], domain 10 [10,49,50,51,52], domain 15 [53,54], domain 20 [52], domain 21 [55], domain 22 [56], domain 23 [57] and domain 24 [5,51,58,59]. Domain 20 is colored green because it is extended and contains an 82 amino acid long insertion. Note that variants denominated as “variant of unknown significance” (VUS) are not listed. ^§^ Recessive mutation; * combination of myopathy and cardiomyopathy; two different mutations at the genetic level. The novel mutations presented in this work are printed red.

With the exception of one described family [42], filaminopathies are caused by dominant pathogenic variants in the *FLNC* gene. The disease is associated with myopathological findings including buildup of protein aggregates and disintegration of sarcomere structures, leading to muscle weakness and dysfunction. To date, 24 different dominant FLNC variants associated with the clinical manifestation of a MFM have been described (Figure 9). We here report on three MFM-filaminopathy patients from two families with novel truncating variants in FLNC: one in exon 30 that encodes part of Ig-like domain 15, and the other in exon 48 encoding the carboxyterminal part of dimerization domain 24 of FLNc. Comprehensive studies were carried out to characterize their clinical and pathogenic consequences.

### 4.1. New FLNC Variants Lead to Selective Skeletal Muscle Involvement in p.Y2704X and an Overlap of Skeletal and Cardiac Alterations in p.Q1662X

In both patients presenting with the p.Y2704X variant, symptom onset with proximal lower limb weakness was diagnosed in sixth decade of life. This coincides with similar descriptions of MFM-filaminopathy families from different countries and with distinct FLNC mutations [60] and thus underlines the largely homogeneous phenotype of this MFM-filaminopathy that is associated with the expression of toxic protein aggregates in skeletal muscle fibers.

Interestingly, patient I:1 (p.Q1662X) with late disease onset and mild skeletal muscle phenotype, also showed cardiac abnormalities, which were initially interpreted as ischemic cardiomyopathy caused by coronary heart disease. However, cardiac MRI revealed structural alterations compatible with a degenerative cardiomyopathy typically seen in FLNc deficiency with a primary cardiac phenotype. This demonstrates an overlap of skeletal muscle and cardiac alterations even in the early disease course and recommends a thorough cardiac follow up in MFM-filaminopathy.

Muscle imaging in the two p.Y2704X patients (II:1 and II:2) revealed that especially semimembranosus, biceps femoris, soleus and anterior tibial muscles showed marked lipomatous changes, while gracilis, sartorius, and the lateral head of the gastrocnemius muscle were relatively spared. This pattern of muscle involvement was also described for other families with MFM-filaminopathy, and thus seems to be typical of MFM-filaminopathy [17,19]. By contrast, MRI in patient I:1 (p.Q1662X) with haploinsufficiency and a shorter disease duration, revealed only focal edematous alterations in the soleus, which are not sufficient for a clear distinction against other neuromuscular disorders. Interestingly, FLNc protein expression is highest in the soleus muscle, at least in mice [40].

The most striking histopathological feature in MFM-filaminopathy is abundant protein aggregation in muscle fibers, with increased immunoreactivity for FLNc and other MFM marker proteins such as Xin. Such aggregates were present in both p.Q1662X and p.Y2704X muscle biopsies, although to a varying degree. While the p.Q1662X skeletal muscle biopsy contained only some muscle fibers with FLNc aggregates and FLNc positive lesions arising from the sarcomeric Z-discs, the p.Y2704X biopsy showed a more pronounced FLNc pathology. It is plausible to assume that this correlates not only with the expression of a toxic, misfolded protein, but also with a longer disease duration and thus a more advanced clinical impairment. 

### 4.2. Not Only the Expression of Toxic FLNc Protein but Also FLNc Haploinsufficiency May Lead to Intracellular Protein Aggregation

FLNc is required to establish and maintain sarcomere structure, integrity, and physiological function. The two novel dominant FLNc variants described here, indicate and confirm that pathogenic variants in FLNC associated with haploinsufficient FLNc expression or expression of a toxic protein can both lead to Z-disc alterations and intracellular accumulation of FLNc and its binding partners in myofibers [53].

Since the p.Y2704X variant is caused by a premature stop codon affecting the last FLNC exon, nonsense mediated decay of the mutant transcript is very unlikely. This is confirmed by the normal amount of FLNC transcripts detected by our RT-PCR experiments, and thus indicates the expression of a truncated, misfolded, and unstable FLNc variant. As demonstrated previously, loss of β-strands F’ and G, caused by the p.W2710X variant, is already sufficient for the inability of Ig-like domain 24 to dimerize, although only one of the two β-sheets is affected (see Figure 6B) [5,39]. It is therefore not surprising that the lack of strands D-G, as found in patients with a p.K2676Pfs*3 mutation, leads to a total loss of the dimerization capability of this domain [61]. the new p.Y2704X mutation now leads to an alteration of the properties of FLNc at the biochemical level that is highly similar to the p.Y2710X mutation: the ability to dimerize in vitro is severely limited, and spontaneous aggregation is displayed. In line with our previous data on a mutation in Ig-like domain 7 [60,62] and the presence of additional MFM-filaminopathy causing variants in other Ig-like domains (Figure 9) [13], this further supports the concept that the mere misfolding of any single Ig-like domain of FLNc is sufficient to cause a phenotype with enhanced lesion and aggregate formation.

The p.Q1662X mutant is also associated with a premature stop codon. Since this stop codon is not localized in the last exon, NMD is expected to be evoked, which we confirmed by our RT-PCR studies. In the majority, such a truncation mutation in FLNC leads to an isolated cardiac phenotype [24,25,57,63], although some patients also have been described to display combined cardiac and skeletal myopathy [49,50]. Most of these patients have a mutation affecting FLNc Ig-like domains 10 or 20 (see Figure 9). It should however not be neglected that also in some of the reported MFM-filaminopathy families, at least a significant fraction of the patients also present with cardiac problems [37]. Our p.Q1662X patient fits into this pattern. The cardiac abnormalities that preceded the presentation of the skeletal muscle phenotype were initially not recognized as filaminopathy associated. A recent cardiac MRI examination however confirmed a cardiomyopathy phenotype typical for filaminopathy patients.

The similarity of the phenotypes of patients with FLNc haploinsufficiency and patients expressing a mutant FLNc protein was noted before, and it was suggested that both kinds of mutations lead to disarray and weakening of Z-discs and cell–cell adhesion sites and thus to an impairment of mechanotransduction [57]. Similarly, it was shown that FLNc haploinsufficient (p.G1674X) cardiomyocytes derived from induced pluripotent stem cells, as well as cells expressing a toxic FLNc protein (p.V1668_G1674del), both established using CRISPR/Cas, both exhibit an increased lysosome expression and activation of autophagic pathways [64]. Thus, it is tempting to speculate that the decreased level of functional FLNc expression observed in both kind of patients causes at least part of the pathomechanisms ultimately eliciting the disease phenotype.

### 4.3. PQS Is Altered Differently in MFM-Filaminopathy Muscle Tissue

UPS and the autophagy-lysosomal pathway are the two major protein degradation systems in eukaryotic cells and are crucial for cellular homeostasis. In skeletal muscle, the general autophagy-lysosomal pathway includes client-specific chaperone-assisted selective autophagy (CASA), a tension-induced autophagy pathway essential for myofibril integrity maintenance by degradation of mechanically damaged FLNc and the simultaneous stimulation of *FLNC* transcription [65].

Prompted by our histopathological findings indicative for the formation of FLNc-positive intracellular aggregates and buildup of autophagic material, transcriptional and immunoblot studies were performed to investigate key PQS pathways in *FLNC* mutant and control (normal and from patients with sIBM) skeletal muscle samples. We hypothesized that as in sIBM, a homogeneous increase in PQS pathways occurs in both FLNC variants to antagonize the accumulation and aggregation of toxic misfolded FLNc protein in intracellular protein deposits. Consistent with this hypothesis, our protein expression studies revealed an increase in all tested PQS markers in sIBM samples. This molecular observation is in line with the known alterations of proteolysis in the pathophysiology of sIBM as a vacuolar myopathy with the presence of intracellular protein aggregations. These alterations may reflect a cellular attempt to elevate protein clearance capacity to facilitate break-down of abnormal protein aggregates in sIBM [66]. In contrast, MFM-filaminopathy samples indicated a clearly distinct pattern of PQS alterations: all three FLNC variants (p.Q1662X, p.Y2704X, and p.W2710X) showed increased BAG3 expression at the protein but not the transcript level (indicating regulation at the post-translational level), with the strongest effect in the patient with the truncated mutation. Protein expression levels of p62 and TRIM32 were, however, strikingly different in these three FLNC variants. In p.Q1662X patient skeletal muscle, pronounced expression of the E3 ubiquitin ligase and UPS marker TRIM32 was evident. Proteolysis in skeletal muscle is closely regulated by E3 ubiquitin ligases such as TRIM32, that was shown to target actin and desmin, and mutations in TRIM32 result in LGMD, nemaline myopathy or MFM. This hints at an activation of the UPS pathway in the early phase of the underlying pathophysiology caused by the p.Q1662X FLNc variant. In the same patient, p62 protein (but not mRNA) level was reduced reinforcing the concept of an activated proteolytic system [67]. This picture changed in protein aggregation-causing mutants: The increased abundance of p62 and TRIM32 protein in p.Y2704X FLNc-mutant skeletal muscles also points to an activation of the proteolytic system, but with less efficiency compared to p.Q1662X FLNC-mutant muscle. Of note, the clinical manifestation of this variant is also milder than of the p.W2710X and p.Y2704X variants. This finding accords with the observed higher amount of intracellular FLNc aggregates in the latter patients compared to the p.Q1662X patient. Indeed, protein expression data in muscle tissue from p.W2710X with a longer disease duration and a drastically higher number of intracellular protein aggregates, also revealed a clearly increased level of the well-known aggregation marker p62, indicating a continuous autophagic buildup with insufficient protein clearance in the MFM-filaminopathy disease course. This is accompanied by a decreased TRIM32 expression which may reflect an exhausted UPS pathway with accumulating FLNc aggregates. Varying activation of proteolytic systems is indicated by increased level of transcripts encoding for proteasomal proteins (LMP7, PSME1 and PSME2), as well as for chaperones (CALR) and key lysosomal players (LAMP1) in p.W2710X FLNC-mutant, but not in p.Y2704X or p.Q1662X FLNc-mutant muscles. Since our findings are based on a limited number of patients, a detailed, quantitative analysis comparing autophagy and UPS marker protein levels in a larger cohort of filaminopathy patients with different categories of *FLNC* mutations is needed to confirm our hypotheses. This may not only yield biomarkers helping to decipher more precisely distinct disease subcategories as well as disease course and severity, but it may also help to define appropriate therapeutic intervention strategies to modulate their activity.

## 5. Conclusions

We have identified two novel truncating *FLNC* variants that are associated with MFM-filaminopathy, but display a varying degree of pathogenicity as reflected by the phenotypical manifestation and the results of combined immunofluorescence, immunoblot and transcript studies. Thus, one may speculate that pathogenic variants leading to a more profound toxic aggregation of proteins (including mutant FLNc itself) are accompanied by an insufficient activation of the proteolytic system and might benefit from therapeutic strategies enhancing or promoting protein folding and clearance capacities.

## Data Availability

Anonymized data not published within this article will be shared upon request from qualified investigators.

## Supplementary Materials

The following supporting information can be downloaded at: https://www.mdpi.com/article/10.3390/cells12091321/s1, Table S1: Antibody and reagent list.
