# Supplementary material for: Novel Filamin C Myofibrillar Myopathy Variants Cause Different Pathomechanisms and Alterations in Protein Quality Systems
Source: Cells. 2023 May 5;12(9):1321. doi: 10.3390/cells12091321 (PMC10177260; doi:10.3390/cells12091321)
Supplement: Supplementary file 1 [file cells-12-01321-s001.zip › cells-2341153-supplementary.pdf]

Supplementary Materials:

Table S1: Antibody and reagent list

Primary antibody	species	dilution	manufacturer, ID / reference
Bag3	rabbit polyclonal	IF 1:2000, WB 1:5000	Abcam, ab47124
CHIP	rabbit polyclonal	1:2000	Calbiochem, PC711
LC3B	rabbit polyclonal	1:200	Cell Signaling, #2775
SQSTM1 / p62 [2C11]	mouse monoclonal	IF 1:1000 , WB 1:1000	Abcam, ab56416
TRIM32	rabbit polyclonal	IF 1:1000, WB 1:1000	Novusbio, NBP1-33737
Ubiquitin	rabbit polyclonal	1:50	Abcam, ab7780
FYCO1	rabbit polyclonal	1:500	Sigma, HPA035526
filamin [RR90]	mouse monoclonal	1:40	[1]
XinA [XC4]	mouse monoclonal	1:1	[2]

Secondary antibody / reagent	species	conjugation	manufacturer, ID
mouse IgG + IgM	goat polyclonal	Alexa Fluor 594	Jackson Immuno-Research, 115-585-068
rabbit	goat polyclonal	Cy3	Jackson Immuno-Research, 111-165-144
mouse IgA	goat polyclonal	Alexa Fluor 488	SouthernBiotech,1040-30
mouse IgG1	goat polyclonal	Alexa Fluor 594	Jackson Immuno-Research, 115-585-205
DNA (nuclei)	DAPI		Thermo Scientific,62248

1. van der Ven, P.F.M.; Obermann, W.M.J.; Lemke, B.; Gautel, M.; Weber, K.; Fürst, D.O. Characterization of Muscle Filamin Isoforms Suggests a Possible Role of γ-Filamin/ABP-L in Sarcomeric Z-Disc Formation. *Cell Motil.* **2000**, *45*, 149–162, doi:10.1002/(SICI)1097-0169(200002)45:2<149::AID-CM6>3.0.CO;2-G.

2. van der Ven, P.F.M.; Ehler, E.; Vakeel, P.; Eulitz, S.; Schenk, J.A.; Milting, H.; Micheel, B.; Fürst, D.O. Unusual Splicing Events Result in Distinct Xin Isoforms That Associate Differentially with Filamin c and Mena/VASP. *Exp. Cell Res.* **2006**, *312*, 2154–2167, doi:10.1016/j.yexcr.2006.03.015.
